# Sequence variation among members of the miR-200 microRNA family is correlated with variation in the ability to induce hallmarks of mesenchymal-epithelial transition in ovarian cancer cells

**DOI:** 10.1186/1757-2215-7-12

**Published:** 2014-01-21

**Authors:** Neda Jabbari, Ashley N Reavis, John F McDonald

**Affiliations:** 1 School of Biology, Parker H. Petit Institute of Bioengineering and Biosciences, 315 Ferst Drive, Atlanta, GA 30332-0363, USA; 2 Integrated Cancer Research Center, Georgia Institute of Technology, 315 Ferst Drive, Atlanta, GA 30332-0363, USA

**Keywords:** Ovarian cancer, Metastasis, MicroRNAs, EMT, MET

## Abstract

**Background:**

Epithelial-Mesenchymal Transition (EMT) is a transient and reversible (Mesenchymal-Epithelial Transition or MET) process by which epithelial cells acquire mesenchymal cell characteristics including reduced intercellular adhesion and increased cell motility. While EMT/MET has long been recognized as an essential component of early embryonic development, there is a growing body of evidence indicating that EMT/MET is also a key component of ovarian cancer (OC) metastasis. Recent findings have implicated members of the miR-200 family of microRNAs (miRNAs) in this process.

**Methods:**

Individual members of the miR-200 family of miRNAs were transiently over expressed in metastatic (mesenchymal-like) OC cell lines. Changes in morphology, molecular profiles and drug sensitivity were monitored relative to cells transfected with a negative control.

**Results:**

Morphological hallmarks of MET were detected in cells transfected with all miR-200 family members. Gene expression profiling demonstrated up regulation of epithelial biomarkers and down regulation of mesenchymal biomarkers in transfected cells although significant variation in molecular response and drug sensitivity was associated with different members of the miR-200 family.

**Conclusions:**

Our results indicate that although ectopic overexpression of all members of the miR-200 family in mesenchymal-like OC cells results in morphological changes characteristic of MET, the underlying molecular changes and induced drug sensitivities are highly variable and correlated with sequence variation within the seed and non-seed regions of individual family members.

## Background

Ovarian cancer is the most lethal of all gynecologic cancers [1]. The majority of OC related deaths is attributable to the spread of cancer cells from the primary tumor to metastatic sites throughout the abdominal cavity [2]. During early stages of metastasis, a subset of primary epithelial tumor cells undergo epithelial-to-mesenchymal transition (EMT), whereby intercellular adhesion complexes are disrupted, the characteristic apico-basal polarity of the cells is lost and cells acquire elevated levels of motility, invasiveness and resistance to standard chemotherapeutic treatments [3-5]. Subsequent to attachment to secondary sites, metastasizing cells undergo a complementary mesenchymal-to-epithelial transition (MET) whereby the metastatic cells reacquire epithelial morphologies and other features characteristic of the primary tumor cells [6]. Because of the high clinical significance of metastasis in ovarian and other cancers, considerable effort is currently being directed towards the development of new classes of agents that may reduce the spread of cancer cells by inducing MET [7].

We have previously shown that mesenchymal-like OC cells undergo MET in response to ectopic over-expression of miR-429, a member of the miR-200 family of miRNAs [8]. This finding is consistent with earlier observations implicating members of the miR-200 family of miRNAs in MET [9,10]. In this paper we report the results of a systematic examination of the effect of ectopic over expression of members of the miR-200 family of miRNAs in OC mesenchymal-like cell lines. The results indicate that although over expression of each member of the miR-200 family induces significant changes in many of the morphological and molecular hallmarks of MET, significant differences exist among family members in the expression of EMT/MET biomarkers and in induced drug sensitivity. This functional variability is associated with sequence variation mapping to both the seed and non-seed regions of individual miRNAs.

## Methods

### Cell culture and miRNA transfection

HEY and HEY A8 ovarian cancer cell lines were provided by Gordon B. Mills (MD Anderson Cancer Center, Houston, TX). SKOV-3 ovarian cancer cells were obtained from the American Type Culture Collection (ATCC, Manassas, VA). Cells were cultured in RPMI 1640 (Mediatech, Manassas, VA) supplemented with 10% FBS (Fetal Bovine Serum; Atlanta Biologicals*,* Lawrenceville, GA) and 1% antibiotic-antimycotic solution (Mediatech-Cellgro, Manassas, VA). For miRNA transfections, 6 × 10^4^ cells were seeded per well in 24-well plates. Cells at exponential phase of growth were transfected with 30 nM miRNA purchased as Pre-miR miRNA Precursors (Ambion, Austin, TX) using Lipofectamine 2000 (Invitrogen, Carlsbad, CA) and according to the manufacturer’s instructions. Cells were allowed to grow for 48 hours before RNA isolation. Ambion Pre-miR miRNA Precursor Negative Control was used as a negative control (nc-miR).

### Image capture and morphological assessment

Morphological changes were monitored using an Olympus IX51 microscope (Olympus Optical, Melville, NY). The effect of treatment on cell morphology was objectively measured using CellProfiler cell-imaging software (2.1.0) [11].

### Quantitative reverse transcription real-time PCR (qRT-PCR)

Total RNA was extracted from cells using the RNeasy mini kit (Qiagen, Valencia, CA). RNA concentrations were measured using a NanoDrop 1000 Spectrophotometer V3.2 (NanoDrop, Wilmington, DE). Highly pure RNA samples (A260/A280 between 2.0 and 2.1) were converted into first-strand cDNA with the Superscript III First-strand Synthesis System (Invitrogen, Carlsbad, CA). Real-time PCR analyses were performed using iQ SYBR Green Supermix (Bio-Rad, Hercules, CA) on the CFX96 real*-*time PCR system (Bio-Rad, Hercules, CA).

The primer sequences employed are as follows: KRT7 (keratin 7): forward 5′- GGACATCGAGATCGCCACCT-3′ and reverse 5′- ACCGCCACTGCTACTGCCA-3′; KRT8 (keratin 8): forward 5′- CCGTGGTTGTGAAGAAGATCG-3′ and reverse 5′-GCTGTTCACTTGGGCAGGAC-3′; KRT18 (keratin 18): forward 5′-TGAGACGTACAGTCCAGTCCTT-3′ and reverse 5′-GCTCCATCTGTAGGGCGTAG-3′; GAPDH: forward 5′- TGCACCACCAACTGCTTAGC -3′ and reverse 5′- GGCATGGACTGTGGTCATGA -3′. The primer sequences for mesenchymal biomarker genes were described previously [8]. The threshold cycle and ΔΔCt method was used for calculating the relative amount of the target RNA. Expression values were normalized using *GAPDH* as a reference gene.

### Immunostaining

5 × 10^3^ cells were cultured on eight well chamber slides. Cells at exponential phase of growth were transfected as indicated above. 48 hours after transfection, media was removed and cells were fixed in 10% neutral buffered formalin for 15 min. Cells were then washed, permeabilized with 0.5% Triton X-100 for 5 min, washed again and blocked with 5% BSA (Bovine Serum Albumin) in PBS for 1 hour. The slides were incubated with mouse monoclonal primary antibodies against CDH1 (E-cadherin) and FN1 (Fibronectin, 1:200; Santa Cruz Biotechnology, Inc., Santa Cruz, CA) in 5% BSA for 1 hour at room temperature. After washing, the slides were then incubated with Alexa Fluor 488 Rabbit Anti-Mouse secondary antibody (1:500, Molecular Probes, Inc., Eugene, OR) for 1 hour at room temperature. After a wash with PBS, counterstaining was performed using DAPI (4′,6-diamidino-2-phenylindole, 1:2000) in PBS for 30 min. Cells were then mounted and expression status of protein biomarkers was assessed using the Zeiss confocal microscope system (Carl Zeiss, Jena, Germany).

### Cisplatin sensitivity

Aliquots of cells were seeded in 96-well plates and treated with nine concentrations of cisplatin ranging from 0.1-50 μM. After 72 hours, TOX-8 reagent (Resazurin based *in vitro* toxicology assay kit, Sigma-Aldrich, St Louis, MO) was added to the wells and fluorescence was measured (λex = 560 nm, λem = 590 nm). Outlier values were removed using Grubbs’ test. The ratio of the background-subtracted fluorescence intensities of drug treated to untreated cultures was calculated in percentages across all concentrations. IC_50_ values were determined by non-linear regression of log-transformed data using a normalized response-variable slope model with GraphPad Prism v.6 (GraphPad Software Inc., La Jolla, CA).

### Statistical analysis

Statistical significance of differences in qRT-PCR experiments between experimental and control samples was determined using a two-tailed Student’s *t*-test. Significance of differences in eccentricity profiles of experimental and control samples was evaluated using Mann-Whitney *U* test. Statistical significance of differences in mean IC_50_ values among miRNA transfections was tested using analysis of variance (ANOVA) and Tukey’s multiple comparison test as a post-hoc test. All experiments were performed using at least three biological replicates.

## Results

### MiR-429 is capable of inducing MET in multiple mesenchymal-like OC cell lines

**Figure 1:**
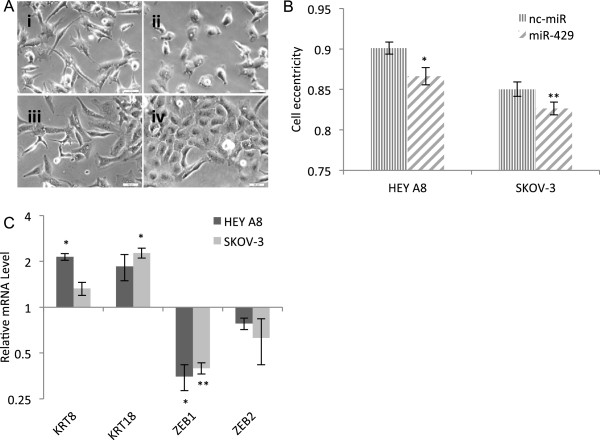
**Over expression of miR-429 induces morphological and molecular changes consistent with the induction of MET. (A)** Representative phase contrast microscopy images of HEY A8 cells transfected with **(i)** nc-miR and **(ii)** miR-429, and SKOV-3 cells transfected with **(iii)** nc-miR and **(iv)** miR-429, 48 hours post transfection. Scale bars, 50 μm. **(B)** The accumulation of rounded epithelial-like cells occurred in miR-429 transfected groups (eccentricity: a score of 0 = circular shape, a score of 1 = linear shape). Values represent mean ± standard error of the mean (SEM). Significance of differences between experimental and control samples is evaluated using Mann-Whitney *U* test (*P <0.05, **P <0.005, n = 127-258 cells analyzed per group). **(C)** Relative mRNA expression of representative epithelial and mesenchymal biomarkers. Expression values in miR-429 transfection are normalized to negative control in each cell line and represent mean ± SEM of three biological replicates each performed in three technical replicates. Asterisks represent significant differences from the negative control (*P <0.005, **P < 0.0005, Student’s *t*-test).

We have previously shown that exogenous over expression of miR-429 induces MET in a well-characterized mesenchymal-like OC cell line (HEY) [8]. We were interested in determining if this ability of miR-429 to induce MET extends to other mesenchymal-like OC cell lines as well. Consistent with our previous results, exogenous over expression of miR-429 in two additional mesenchymal-like OC cell lines (HEY A8 [12] and SKOV-3 [13]) was found to induce morphological and molecular changes consistent with MET (Figure 1). Both SKOV-3 and HEY A8 cells display a change from the elongated shape characteristic of mesenchymal cells to the more cuboidal shape characteristic of epithelial cells after transfection with miR-429. This morphological change was objectively validated using the CellProfiler cell image analysis software [11]. Changes in expression of two epithelial KRT8 and KRT18 and two mesenchymal [ZEB1 and ZEB2 (zinc finger E-box binding homeobox 1 and 2)] molecular biomarkers were consistent with MET.

### All members of the miR-200 family of miRNAs are capable of inducing MET in OC mesenchymal-like cells

**Figure 2:**
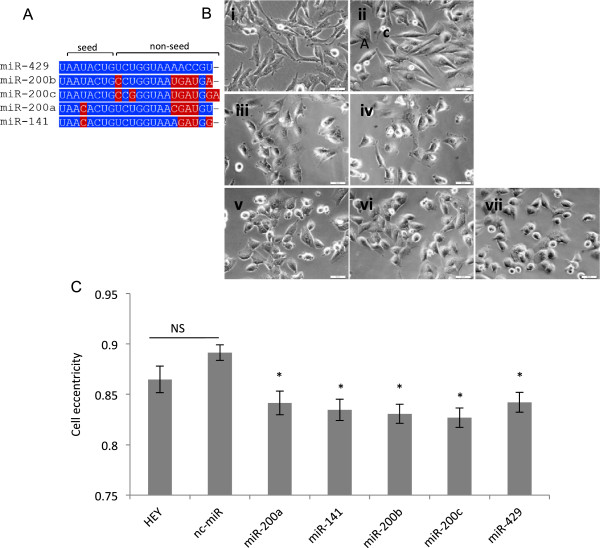
**Over expression of the miR-200 family induces morphological changes consistent with the induction of MET. (A)** Multiple sequence alignment of the miR-200 family. Alignment is performed using the ClustalW2 program. Sequence differences relative to miR-429 are highlighted. **(B)** Representative phase contrast microscopy images of **(i)** HEY cells (not transfected), HEY cells transfected with **(ii)** nc-miR, **(iii)** miR-200a, **(iv)** miR-141, **(v)** miR-200b, **(vi)** miR-200c, and **(vii)** miR-429, 48 hours post transfection. Scale bars, 50 μm. **(C)** The accumulation of rounded epithelial-like cells occurred in miR-200 family transfected cells. Values represent mean ± SEM (n = 124-211 cells analyzed per group). Asterisks represent significant differences from the negative control (nc-miR) (*P <0.0001, NS = not significant, Mann-Whitney *U* test).

MiR-429 is a member of the miR-200 family of miRNAs (Figure 2A). All members of the miR-200 family have previously been either directly or indirectly implicated in EMT [9,10]. Having established that ectopic over expression of miR-429 induces MET in a variety of mesenchymal-like OC cell lines, we were interested in determining if this MET-inducing potential extends to other members of the miR-200 family as well.

The results presented in Figure 2 demonstrate that over expression of all miR-200 family members results in a significant change from the elongated, spindle-shaped morphology of the mesenchymal-like cells to the more rounded, cuboidal morphology characteristic of epithelial cells. No detectable change in morphology was observed in cells treated with the negative control (nc-miR).

**Figure 3:**
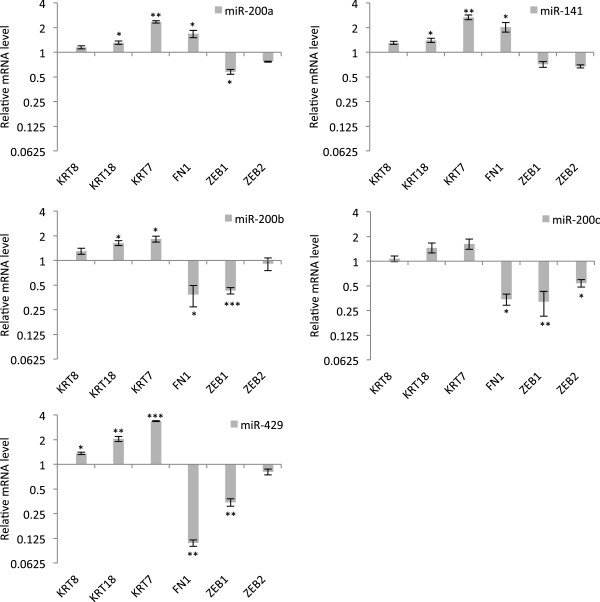
**Over-expression of the miR-200 family induces changes in biomarkers consistent with the induction of MET.** Changes in the expression of representative epithelial and mesenchymal biomarkers after over-expression of miR-200 family members in HEY cells. Expression values are normalized to nc-miR transfected cell and represent mean ± SEM of at least three biological replicates each performed in three technical replicates. Asterisks represent significant differences from the negative control group (*P <0.05, **P <0.005, ***P <0.0005, Student’s *t*-test).

The expression levels (qRT-PCR) of a series of previously established epithelial and mesenchymal biomarkers [14] were monitored in HEY cells transfected with members of the miR-200 family. Consistent with acquisition of a more epithelial phenotype, expression levels of all of the epithelial biomarkers (KRT8, KRT18, KRT7) were increased after over expression of each member of the miR-200 family (Figure 3). As expected, the mesenchymal biomarkers ZEB1/ZEB2 displayed a general reduction in expression levels after over expression of each member of the miR-200 family. The mesenchymal biomarker FN1 was also significantly down regulated in cells transfected with miR200b, miR200c and miR429.

**Figure 4:**
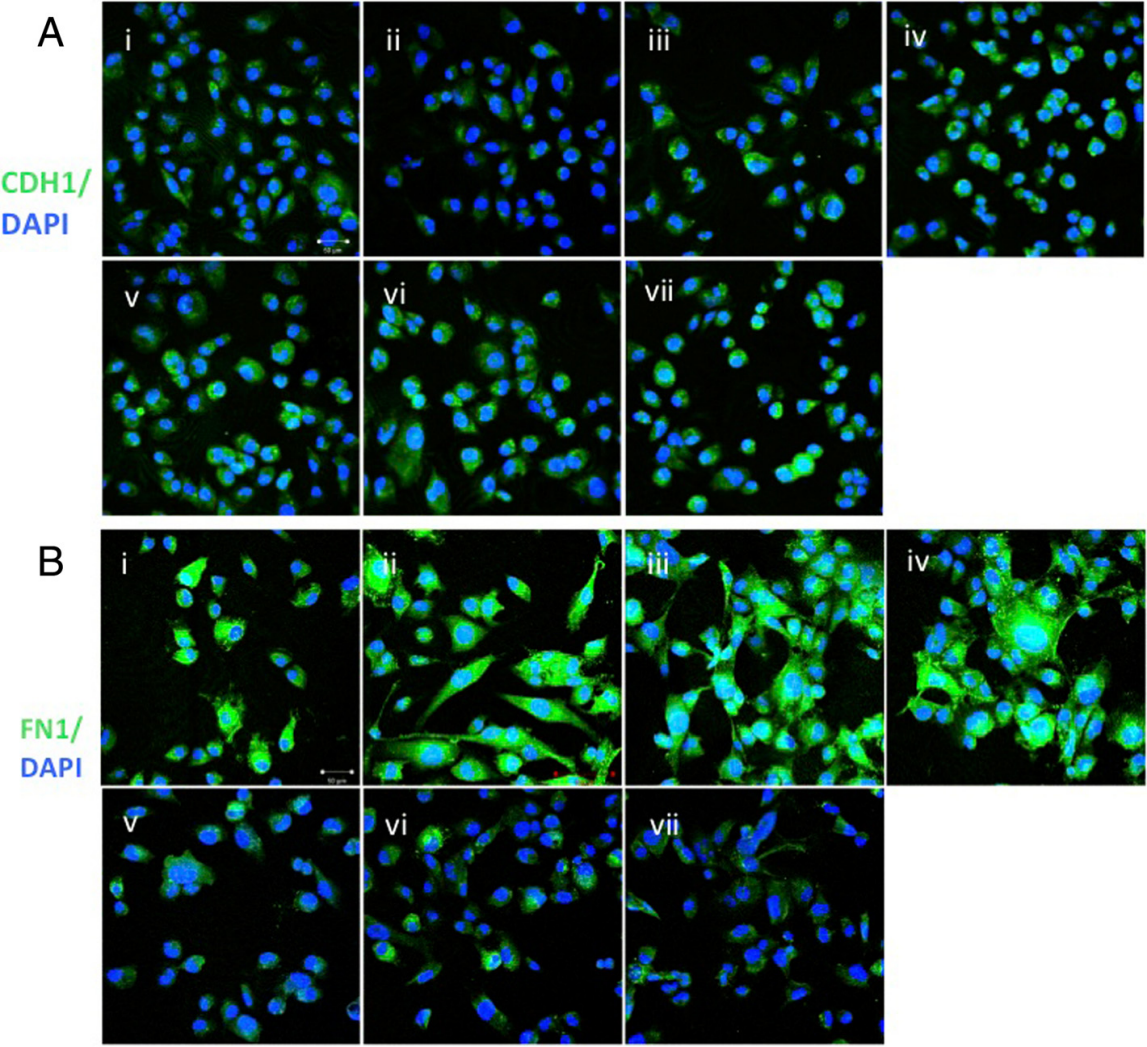
**Immunostaining of cells for CDH1 (A) and FN1 (B) proteins. (i)** HEY cells (not transfected), HEY cells transfected with **(ii)** nc-miR, **(iii)** miR-200a, **(iv)** miR-141, **(v)** miR-200b, **(vi)** miR-200c, and **(vii)** miR-429. DAPI was used to stain the cell nuclei. Scale bars, 50 μm.

Human miRNAs are known to be capable of regulating expression of their target genes by modulating mRNA levels and/or blocking translation [15]. Thus, to further explore the inconsistent response of changes in FN1 RNA levels after transfection by individual members of miR200 family, we additionally monitored expression changes on the protein level by immunofluorescence staining. The epithelial biomarker, CDH1 [14,16], was included in these assays as an additional control (Figure 4).

Consistent with the observed increase in expression levels of each of the epithelial biomarkers monitored on the RNA level, CDH1 protein was detected in all transfected cells but was absent in un-transfected cells and in cells transfected with the negative control. Interestingly, levels of FN1 protein were generally consistent with what was observed on the RNA level, *i.e*., protein levels were significantly down regulated in cells transfected by miR-200b, miR-200c or miR-429 but unchanged or slightly up-regulated in cells transfected by miR-200a, miR-141 (Figure 4). Collectively, these findings suggest that FN1 is a regulatory target of miR-200b, miR-200c and miR-429 but is not subject to regulation by miR-200a and miR-141. In addition, since a change from a mesenchymal to an epithelial phenotype was observed in cells transfected by all members of the miR-200 family, we conclude that FN1 is non-essential for MET in OC.

Previous studies indicate that the most functionally significant component of miRNA/mRNA pairing involves the miRNA “seed region”, *i.e*., nucleotides 2-8 on the 5′ end of the miRNA [17]. Although all members of the miR-200 family are sequentially distinct, miR-200b, miR-200c and miR-429 are sequentially identical within their respective seed regions (Figure 2A). This identity may explain their shared ability to down regulate FN1 when ectopically over expressed in HEY cells. To further explore this possibility, we utilized three miRNA target prediction algorithms (miRanda [18], miRDB [19], and TargetScan [20]) to identify members of the miR-200 family that are predicted to directly target FN1 mRNA and the other biomarkers monitored in our PCR experiments. The results, presented in Table 1, indicate that all three algorithms predict the presence of binding sites in FN1 mRNA for miR-200b, miR-200c and miR-429, but not for miR-200a, miR-141. These computational predictions are consistent with our experimental results and indicate that differences in target specificity for FN1 mRNA between the miR-200 family members accounts for the observed variation in regulation of FN1 expression.

**Table 1:** Prediction of miR-200 binding sites on FN1, ZEB1, ZEB2, KRT7, KRT8 and KRT18 mRNAs

**miRNA**	**miR-200b**			**miR-200c**			**miR-429**			**miR-200a**			**miR-141**		
**Prediction algorithm**	†A	¥B	ǂC	A	B	C	A	B	C	A	B	C	A	B	C
**FN1**	2	2	1	2	2	1	2	2	1	0	0	0	0	0	0
**ZEB1**	5	6	5	5	6	5	5	6	5	3	3	3	3	3	3
**ZEB2**	6	5	6	6	5	6	6	5	6	3	4	2	3	4	2
**KRT7**	0	0	0	0	0	0	0	0	0	0	0	0	0	0	0
**KRT8**	0	0	0	0	0	0	0	0	0	0	0	0	0	0	0
**KRT18**	0	0	0	0	0	0	0	0	0	0	0	0	0	0	0

Only those genes predicted to be targeted by miR-200 family members were observed to be downregulated in cells in which they were ectopically overexpressed.

†A: miRanda (mirSVR ≤ -0.2), ¥B: miRDB, ǂC: TargetScan (conserved miRNA sites).

### HEY cells transfected by miR-200 family members display significant variation in sensitivity to cisplatin associated with variation within the non-seed region of individual miRNAs

Previous studies indicate that EMT is often associated with decreased sensitivity of a variety of cancer cells to chemotherapy [21-23]. Consistent with this observation, OC cells undergoing EMT have been reported to display a decreased sensitivity to platinum-based drugs, a frequently employed first line therapeutic in the treatment of OC [24,25]*.* Since EMT is reported to decrease the sensitivity of OC epithelial cells to platinum-based drugs, we were interested in determining if miR-induced MET of mesenchymal-like OC cells would induce an opposite effect, *i.e.*, be associated with an increased sensitivity of OC cells to platinum-based drugs. To explore this possibility, we monitored the drug susceptibility (IC_50_) of HEY cells to cisplatin after miRNA-induced MET relative to controls.

**Figure 5:**
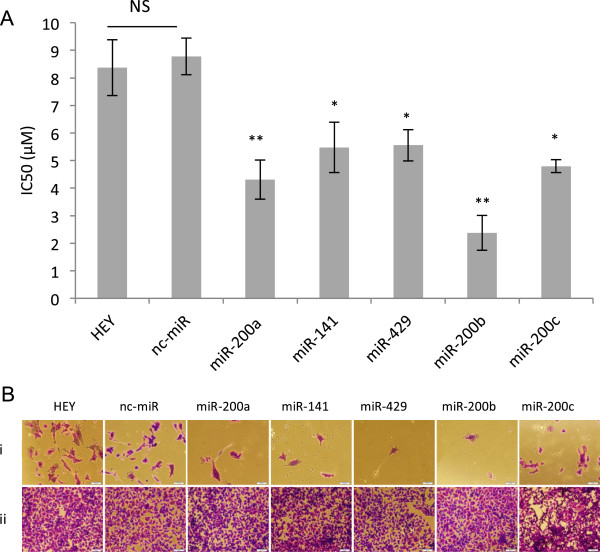
**Effect of over expression of members of miR-200 family in HEY cells on cisplatin sensitivity. (A)** IC_50_ values are presented as mean ± SEM of at least 3 independent experiments with 3 replicated wells per drug concentration. Statistical analysis was performed using ANOVA and Tukey’s multiple comparison test as a post-hoc test. Asterisks represent significant differences from the negative control (nc-miR) group, *P ≤0.05, **P ≤0.005. **(B)** Qualitative evaluation of cell viability in presence of **(i)** 20 μM and **(ii)** 0 μM cisplatin in HEY cells alone or transfected with different members of miR-200 family or nc-miR (crystal violet staining; Scale bars, 100 μm).

The results presented in Figure 5 demonstrate that ectopic over expression of all members of the miR-200 family results in a significant increase in cisplatin sensitivity relative to negative control. While notable variation was observed among cells transfected by different members of the miR-200 family, in most cases this variation was not statistically significant. Cells transfected with miR-200b were significantly more sensitive to cisplatin than those transfected with miR-429 (p < 0.05). Interestingly, these two miRNAs share identical seed regions suggesting that non-seed sequences may be of significance in miR-200b induced drug sensitivity.

## Discussion

It is widely acknowledged that the metastatic spread of cancer cells is responsible for most cancer related deaths [26,27]. This is particularly true for OC where the prospect of favorable outcome drops precipitously once cancer cells migrate beyond the confines of the primary tumor [28]. Since the EMT/MET process is believed to play a central role in the metastasis of many cancers [29] including OC [30,31], considerable effort is being focused on the discovery and development of reagents that may intervene in this process for therapeutic benefit [7].

We previously demonstrated that ectopic over expression of miR-429, a member of the miR-200 family of microRNAs, is sufficient to convert highly metastatic OC mesenchymal-like cells to epithelial phenotype with a concomitant reduction in invasive and migratory potentials [8]. Prompted by these initial results, we were interested in determining if the MET-inducing properties of miR-429 extended to other OC mesenchymal-like cell lines and if other members of the miR-200 family were also capable of exerting these potentially anti-metastatic effects. Finally, since previous studies have shown that OC cells induced to undergo EMT display a significantly decreased sensitivity to platinum-based drugs [24,25], we were interested to determine if miRNA-induced reversal of the process (*i.e*., MET) might increase sensitivity to these drugs thereby demonstrating an additional potential therapeutic benefit of these small regulatory RNAs.

Consistent with our earlier studies, we found that ectopic over expression of miR-429 in two additional OC mesenchymal-like cell lines (SKOV-3 and HEY A8) results in morphological and molecular changes characteristic of MET. Ectopic over expression of other members of the miR-200 family in mesenchymal-like HEY cells also induced morphological changes characteristic of MET but underlying molecular changes were found to be variable and attributable to sequence variation within the seed region of individual family elements. Finally, we tested the sensitivity of HEY cells to cisplatin after transfection with members of the miR-200 families relative to negative controls. The results demonstrate that although miR-200 family induced MET is generally correlated with a significant increase in sensitivity to cisplatin, significant variation exists among individual family members. In this case, the variability was associated with sequence variation mapping to the non-seed region of individual family members.

## Conclusions

Collectively our results are consistent with earlier findings from our lab [8] and others [9,10] indicating that members of the miR-200 family are involved in EMT/MET and OC metastasis. Our results also indicate that significant variation exists among family members in the regulation of molecular processes underlying specific features of the EMT/MET process and that this variation is associated with sequence variation mapping to both the seed and non-seed region of individual family members. Our results generally support the notion that exogenous modulations in the expression of miRNAs involved in EMT may serve as the basis of important new strategies in the treatment of ovarian and other types of cancer [32].

## Abbreviations

EMT: Epithelial-mesenchymal-transition; MET: Mesenchymal-epithelial-transistion; miRNAs: MicroRNAs; nc-miR: Negative control miRNA; OC: Ovarian cancer.

## Competing interests

The authors declare that they have no competing interests.

## Authors’ contributions

JM and NJ conceived the study and wrote the paper. NJ and AR performed the experiments. NJ carried out the statistical analyses. All authors read and approved the final manuscript.
